# Generation of a Biobank From Two Adult Thoroughbred Stallions for the Functional Annotation of Animal Genomes Initiative

**DOI:** 10.3389/fgene.2021.650305

**Published:** 2021-03-08

**Authors:** Callum G. Donnelly, Rebecca R. Bellone, Erin N. Hales, Annee Nguyen, Scott A. Katzman, Ghislaine A. Dujovne, Kelly E. Knickelbein, Felipe Avila, Ted S. Kalbfleisch, Elena Giulotto, Nicole B. Kingsley, Jocelyn Tanaka, Elizabeth Esdaile, Sichong Peng, Anna Dahlgren, Anna Fuller, Michael J. Mienaltowski, Terje Raudsepp, Verena K. Affolter, Jessica L. Petersen, Carrie J. Finno

**Affiliations:** ^1^Department of Population Health and Reproduction, School of Veterinary Medicine, University of California, Davis, Davis, CA, United States; ^2^Veterinary Genetics Laboratory, School of Veterinary Medicine, University of California, Davis, Davis, CA, United States; ^3^Morris Animal Foundation, Denver, CO, United States; ^4^Department of Surgical and Radiological Sciences, School of Veterinary Medicine, University of California, Davis, Davis, CA, United States; ^5^Gluck Equine Research Center, University of Kentucky, Lexington, KY, United States; ^6^Department of Biology and Biotechnology, University of Pavia, Pavia, Italy; ^7^Department of Animal Science, University of Nebraska-Lincoln, Lincoln, NE, United States; ^8^Department of Animal Science, College of Agricultural and Environmental Sciences, University of California, Davis, Davis, CA, United States; ^9^Department of Veterinary Integrative Biosciences, Texas A&M University, College Station, TX, United States; ^10^Department of Pathology, Microbiology, and Immunology, School of Veterinary Medicine, University of California, Davis, Davis, CA, United States

**Keywords:** FAANG, horse, male, stallion, biobank

## Abstract

Following the successful creation of a biobank from two adult Thoroughbred mares, this study aimed to recapitulate sample collection in two adult Thoroughbred stallions as part of the Functional Annotation of the Animal Genome (FAANG) initiative. Both stallions underwent thorough physical, lameness, neurologic, and ophthalmic (including electroretinography) examinations prior to humane euthanasia. Epididymal sperm was recovered from both stallions immediately postmortem and cryopreserved. Aseptically collected full thickness skin biopsies were used to isolate, culture and cryopreserve dermal fibroblasts. Serum, plasma, cerebrospinal fluid, urine, and gastrointestinal content from various locations were collected and cryopreserved. Under guidance of a board-certified veterinary anatomic pathologist, 102 representative tissue samples were collected from both horses. Whole tissue samples were flash-frozen and prioritized tissues had nuclei isolated and cryopreserved. Spatially contemporaneous samples of each tissue were submitted for histologic examination. Antemortem and gross pathologic examination revealed mild abnormalities in both stallions. One stallion (ECA_UCD_AH3) had unilateral thoracic limb lameness and bilateral chorioretinal scars. The second stallion (ECA_UCD_AH4) had subtle symmetrical pelvic limb ataxia, symmetrical prostatomegally, and moderate gastrointestinal nematodiasis. DNA from each was whole-genome sequenced and genotyped using the GGP Equine 70K SNP array. The genomic resources and banked biological samples from these animals augments the existing resource available to the equine genomics community. Importantly we may now improve the resolution of tissue-specific gene regulation as affected by sex, as well as add sex-specific tissues and gametes.

## Introduction

Sex bias in animal model-based biomedical research has been a focus area for the National Institutes for Health (NIH) (Will et al., 2017). While the influence of sex on experimental outcomes is undeniable, the adoption of sex as a consideration for experimental design has been slow (Zucker and Beery, 2010). Genomic regulation is particularly labile to the effect of sex (Lee, 2018). With the intent of cataloging the functional and regulatory elements of economically significant animal species, there is need for the Functional Annotation of Animal Genome (FAANG) project to diligently account for sex differences. This is of importance to the equine industry in which sex of animal is an important component of management and use, such as predominance of females in polo competition and males in show jumping (Fenner et al., 2019). The increased resolution of tissue specific genomic, transcriptional, and epigenomic data will be of significant benefit to the progress of equine genomics. In particular, the ability to more precisely characterize regulation of genomic regions associated with traits pertinent to equine health and performance are made increasingly more possible (Kingsley et al., 2019). The objective of the current study was to augment the existing equine biobank with male samples. We report here the addition of two male Thoroughbred horses to the biobank of two female Thoroughbred horses initially established in 2016.

## Materials and Methods

### Animals

Two Thoroughbred stallions (aged 3 and 4 years; ECA_UCD_AH3 and ECA_UCD_AH4, respectively) were donated for this project. ECA_UCD_AH3 had been in racing training and sustained a career ending musculoskeletal injury prior to donation. ECA_UCD_AH4 had not engaged in racing training, and is a son of the reference genome donor Twilight. Approval for all protocols was granted by the UC Davis Institutional Animal Care and Use Committee (Protocol #21033).

### Clinical Assessments

Complete physical examination (including complete blood count and serum biochemistry) was performed and interpreted by two board-certified internists (CJF and CGD) for both stallions. Complete neurologic and lameness examinations were performed by relevant recognized experts (CJF and SAK, respectively) for both stallions within 24 h prior to euthanasia. Complete ophthalmic examination of the anterior and posterior segment (performed by KEK) as well as photopic and scotopic electroretinography (ERG) was performed on both stallions within 48 h prior to euthanasia.

### Clinical Sample Collection

Serum and plasma samples were collected immediately prior to euthanasia *via* an indwelling intravenous cannula. Heparin and EDTA whole blood was centrifuged at 2,000× g for 10 min at 4°C. Plasma was harvested, flash frozen in liquid nitrogen and stored at −80°C. Whole blood in plain tubes was allowed to clot for 30 min at room temperature, with serum collected in the same fashion as plasma.

Ejaculated sperm and seminal plasma were collected from ECA_UCD_AH3 at the time of ophthalmic examination. Ejaculated sperm was unable to be collected from ECA_UCD_AH4. Seminal plasma was retrieved by un-cushioned centrifugation at 2,000× g for 15 min. The supernatant was separated and centrifuged again before being passed through a 20 μm filter. The supernatant and sperm pellet were flash frozen in liquid nitrogen and stored at −80°C.

Immediately following euthanasia, the left testicle, epididymis, and distal ductus deferens were removed from each horse. The epididymes were removed and incised sharply transversely at the cauda epididymis. Commercial semen extender was flushed retrograde *via* the remaining ductus deferens. Recovered spermatozoa were frozen in 0.5 mL polyethylene straws by controlled rate freezing protocol and stored in liquid nitrogen, at 323 and 455.5 million sperm/mL for ECA_UCD_AH3 and ECA_UCD_AH4, respectively.

Synovial fluid was collected aseptically (by centesis) from the left middle carpal, radio-carpal joint and the left medial compartment of the femoro-tibial joint from ECA_UCD_AH3 and the right middle carpal, radio-carpal joint and left medial compartment of the femoro-tibial joint from ECA_UCD_AH4 immediately following euthanasia. A veterinary clinical pathologist cytologically evaluated an aliquot of each sample within 2 h of collection. The remaining sample was centrifuged at 2,000× g for 10 min at 4°C, and the supernatant was flash frozen in liquid nitrogen and stored at −80°C.

Cerebrospinal fluid was collected aseptically *via* atlanto-occipital centesis. A veterinary clinical pathologist cytologically evaluated an aliquot of each sample within 2 h of collection. The remaining sample was then processed in the same manner as synovial fluid.

### Peripheral Blood Mononuclear Cell Collection

Whole blood was collected in EDTA tubes 24 h prior to humane euthanasia. Using density gradient centrifugation, peripheral blood mononuclear cells (PBMCs) were harvested as previously described (Hida et al., 2002). Briefly, whole blood was overlayed on a double gradient of 1,077 and 1,119 histopaque media (Sigma-Aldrich). Then, samples were centrifuged at 700 g for 30 min with the enriched PBMC layer carefully removed, flash frozen in liquid nitrogen and stored at −80°C.

### Tissue Specific Sampling

Full thickness skin biopsies were aseptically collected from the area overlying the left gluteal muscles for dermal fibroblast isolation and cultured as previously described (Raimondi et al., 2011). Briefly, biopsies were washed in ice cold PBS with the addition of penicillin and streptomycin. Fragments of the dermis (2–3 mm^2^) were placed into a 24-well tissue-culture-treated plate and covered in complete media (Dulbecco's Minimum Essential Medium, 20% fetal bovine serum, 2× non-essential amino acids, 2 mM L-glutamine, 2× penicillin/streptomycin, 2 μg/ml amphotericin B, and 1 μg/ml fluconazole). At confluence, cells were trypsinized, counted, and seeded in a 12-well plate. Cells were passaged and frozen at passage three and four in a DMSO-based cryoprotectant media and stored in liquid nitrogen (Raimondi et al., 2011). DNA isolated from whole blood and from cultured fibroblast cells of each horse were genotyped and compared for 15 standard genetic markers (fourteen microsatellites and one sex link marker amelogenin) routinely tested for use in identify and parentage testing services as the UC Davis Veterinary Genetics Laboratory.

A total of 102 issue samples were taken from all body systems (Supplementary Table 1). Sample stations were arranged in teams, with a veterinarian overseeing each team to ensure appropriate tissue identification. Additionally, all tissues were examined by a veterinary anatomic pathologist (VKA) for gross abnormalities prior to collection (Supplementary Table 2). Tissue samples were collected as previously described (Burns et al., 2018). Briefly, samples from the most representative portion of each tissue were collected and preserved in 10% buffered formalin for histopathology. Samples for nuclei preparation and tissue banking were taken from sites immediately adjacent (proximal and distal) to the representative histopathology sections and flash frozen. Nuclei isolation was performed as similarly to that described for the mare biobank, with the exceptions of testis replacing ovary and C6 spinal cord instead of T1 (Burns et al., 2018).

### Genotyping and Whole-Genome Sequencing

Genomic DNA was isolated from whole blood using a previously validated technique (Burns et al., 2018). Whole-genome sequencing using an Illumina NovaSeq (150 bp paired-end reads) platform was performed at Admera Health, LLC (South Plainfield, NJ), targeting 35X coverage for each horse. Library preparation was performed using the KAPA library quantification kit for Illumina (Roche Holding AG, Basel, Switzerland). SNP genotyping was performed using the GGP Equine 70K SNP bead chip array (Neogen GeneSeek, Lincoln, NE). Genotype data were merged with those collected from the prior sampling effort of Thoroughbred mares (Burns et al., 2018). Marker positions were translated from EquCab2 to EquCab3 using the NCBI Genome Remapping Service (https://www.ncbi.nlm.nih.gov/genome/tools/remap), removing markers that were not mapped to any of the 31 autosomes. Using SNP data, runs of homozygosity (ROH), a measure of diversity, was quantified in DetectRuns (https://github.com/bioinformatics-ptp/detectRUNS/tree/master/detectRUNS) using the method of Marras et al. (2015). Analysis parameters required a minimum of 15 SNPs, maxOpp Run = 1, max MIssRun = 1, maxGap = 10,000,000, minLengthBps = 100,000. Whole-genome sequence data of the stallions and mares were processed and variants called according to the pipeline outlined in Sieck et al. (2020), with the exception of mapping to the EquCab3 reference genome (Burns et al., 2018; Sieck et al., 2020). Bi-allelic autosomal SNPs were extracted from the resulting vcf for ROH analysis in the same manner as used for the SNP data.

Additionally, in an effort to provide extensive phenotyping and genotyping for future studies on pigmentation biology, both stallions were genotyped for coat color loci using the commercially available horse full coat color and white pattern panel (https://vgl.ucdavis.edu/panel/full-coat-color-pattern-panel).

### Pedigree Analysis

Individual inbreeding coefficients (F) were calculated from five-generation pedigrees of both stallions and the previously sampled mares (Burns et al., 2018) using Pedigraph (Garbe and Da, 2008).

### Karyotyping

Sodium heparin stabilized whole blood samples were collected 7 days prior to euthanasia for karyotype characterization. Karyotypes were generated for each horse using the previously validated Pokeweed-stimulated lymphocyte culture (Raudsepp et al., 2010). Thirty metaphase cells from ECA_UCD_AH3 and ECA_UCD_AH4 were captured for analysis. Genomic DNA was isolated from lymphocyte cultures for PCR detection of the sex determining region Y *(SRY)* and androgen receptor *(AR)* genes, as preciously described (Raudsepp et al., 2004).

## Results

### Clinical Assessments

ECA_UCD_AH3 had several mild abnormalities apparent on clinical examination, including a grade III/V left thoracic limb lameness [AAEP scale (Baxter, 2020)] and chorioretinal scarring of both eyes. Scoptopic ERG data for both horses is provided in Supplementary Table 3. No other abnormalities were detected antemortem for ECA_UCD_AH3. ECA_UCD_AH4 did not have clinically detectable lameness, however, a mild grade I/V [Modified Mayhew scale (Furr and Reed, 2015)] symmetrical pelvic limb ataxia was detected with a presumptive neuroanatomical localization to the cervical spinal cord. No other abnormalities were detected for ECA_UCD_AH4 on clinical examination. No abnormalities were detected on complete blood count or serum biochemistry for either stallion.

### Clinical Sample Collection

Serum, plasma (collected in EDTA and heparin tubes), buffy coat and urine were collected from both stallions. Seminal plasma and ejaculated spermatozoa were obtained for ECA_UCD_AH3 only. Epididymal recovered spermatozoa from both stallions were successfully isolated and cryopreserved with adequate post thaw kinematic parameters (Supplementary Table 4). Synovial and cerebrospinal fluid samples were cytologically normal (Supplementary Tables 5, 6). PBMCs were successfully collected from both stallions.

### Genotyping

Achieved coverage of the whole-genome sequence data was 38.7 and 37.5X for ECA_UCD_AH3 and ECA_UCD_AH4, respectively. Relative to the reference genome, between 5.4 (ECA_UCD_AH3) and 3.8 (ECA_UCD_AH4) million variants were observed. Data are publically available through the FAANG consortium. Combining the 70K genotype data of the stallions to that from the two mares and removing variants present on sex chromosome data and on unmapped contigs, 59,823 variants remained. The count of ROH per horse was similar (498–503) for all four horses, except ECA_UCD_AH4 (280). However, ECA_UCD_AH4 had a greater number of long (>8 Mb) ROH than all other horses (Supplementary Table 7). Considering SNP data from WGS, between 1,822 (ECA_UCD AH1) and 2,867 (ECA_UCD AH4) ROH were observed. Five-generation pedigree-based inbreeding estimates ranged from 0 (ECA_UCD AH1) to 0.344 (ECA_UCD AH4).

Genetic profiles from cultured fibroblast cells matched to that of the expected horse (15/15 markers matched for each horse, presented in Supplementary Table 8).

ECA_UCD_AH3 is genetically defined as a bay horse (E/e A/a) with one copy of dominant white 20 (N/W20), while ECA_UCD_AH4 was gray with a bay base coat (E/e A/A) before graying out and was homozygous for dominant white 20 (W20/W20) (all coat color genotypes are presented in Supplementary Table 9).

### Karyotype

Both stallions had a normal 64 XY male karyotype with no apparent structural or numerical chromosomal abnormalities. Both stallions were positive for the *SRY* and *AR* genes.

### Tissue Specific Sampling

Sample collection commenced 30 min following euthanasia and was concluded within 3 h for all tissue samples. Samples from 102 tissues were collected and flash frozen (Supplementary Table 1). Sixteen of these tissues also had nuclei isolated and cryopreserved (Supplementary Table 1).

### Pathology

Gross examination of ECA_UCD_AH3 was without abnormality, except for a 15 × 2 × 1.3 cm firm area at the caudal margin of the left caudal lung lobe. This area was therefore avoided for sampling. Histologic examination of the area identified locally extensive fibrosis, Type II pneumocyte proliferation and alveolar histiocytosis, which is consistent with a previous pulmonary inflammatory insult. Mild lymphocytic inflammation was noted at several sections of the gastrointestinal tract, but were considered incidental. Gross examination of ECA_UCD_AH4 revealed marked gastrointestinal nematodiasis throughout the large and small intestinal segments. Gastrointestinal segments had marked lymphocytic and eosinophilic infiltrates consistent with gastrointestinal parasitism. ECA_UCD_AH4 had marked prostatomegally grossly, but was without abnormality on histopathology. There were no gross or histologic findings of the central nervous system of ECA_UCD_AH4 that could explain the mild sensory ataxia. There were no other significant histologic abnormalities. Summary of all pathologic findings are summarized in Supplementary Table 2.

## Discussion

We report the successful completion of the male equine biobank for the FAANG consortium. This project has added 102 tissues from two stallions in addition to seven body fluids, one cell line and spermatozoa to the biobank for use by the equine research community. This study recapitulated the guidelines of the original equine tissue collection, demonstrating that this method is repeatable and appropriate for this type of endeavor.

Stringent phenotype information both ante- and postmortem are a critical feature of this biobank and lends strength to the interpretation of results of subsequent studies using these tissues. Similarly, it also establishes the boundaries for the interpretation of data derived from this biobank. This is especially true for the gastrointestinal tissues that had lymphocytic and/or eosinophilic infiltrates. Previous tissue collection from both mares also had a similar infiltrate pattern in the gastrointestinal sections to the stallions described here (Burns et al., 2018). While this makes the direct comparison between male and female samples more consistent, it underscores the necessity to perform detailed phenotypic characterization of the tissues. The available phenotype information was expanded in this data set with the addition of ERG measurements. Further, this additional information enhances the ability to select tissue with the most normal function or, at minimum, document pathology that may affect future assays. In this case, the left retina from ECA_UCD_AH3 with an abnormal waveform was not used for the tissue bank, with the normal right retina retained.

The available whole-genome sequencing and SNP genotyping data from these horses to accompany the biobank and pending tissue-specific regulatory information will assist in future genomic investigations. In this sampling effort, the availability of a stallion closely related to the mare from which the reference genome is based (Twilight) is expected to provide high accuracy mapping and allow for more in depth investigations of achieved vs. predicted inbreeding. The intentional inbreeding that resulted in ECA_UCD_AH4 (by breeding record) was corroborated by the frequency and relatively large ROH as identified in the analysis of the SNP genotype data. This also highlights the limitation of this biobank, as it is restricted to a single breed. Future investigation may need to include more diverse biologic replicates to account for between breed variations in gene regulation (Li et al., 2012).

While the equine FAANG project has not progressed to the level of the ENCODE project in humans, important data have already been generated from the equine biobank (ENCODE Project Consortium, 2012). Histone modifications as analyzed by chromatin immunoprecipitation sequencing have been established for eight tissues from the original two mares (Kingsley et al., 2019). The addition of repressive and enhancing epigenetic marks to the catalog of equine genome regulation is of great benefit to the equine research community. Sex-biased gene regulation is strongly intertwined with epigenetic regulation, especially as it relates to histone modifications (Tsai et al., 2009). This reinforces the need for the equine FAANG project to expand the existing biobank with male samples. This undertaking was no small feat and required the sacrifice of two additional animals. However, the benefit to future equine genomic studies is immense.

Although this study concludes the tissue collection arm of the FAANG project, the work to annotate the equine genome continues in earnest. This addition to the biobank expands the list of tissues available to the equine research community. Groups with specific interest in certain tissues are encouraged to contact the corresponding author for availability, as the “adopt-a-tissue” program successfully used with the original collection is also available for these male samples.

## Data Availability Statement

The datasets presented in this study can be found in online repositories. The names of the repository/repositories and accession number(s) can be found at: https://www.ebi.ac.uk/ena, PRJEB42407.

## Ethics Statement

The animal study was reviewed and approved by UC Davis Institutional Animal Care and Use Committee.

## Author Contributions

Clinical examinations were performed by CF, SK, KK, and CD. Semen evaluation and cryopreservation performed by GD. Karyotyping performed by TR. Tissue collection performed by EH, AN, KK, NK, JT, EE, SP, AD, AF, JP, and CD. All histopathoogy reviewed by VA. Nuclei preparation performed by RB and FA. Cell culture and cryopreservation performed by MM. Genetic analysis performed by JP and RB. All authors contributed to manuscript preparation.

## Conflict of Interest

The authors declare that the research was conducted in the absence of any commercial or financial relationships that could be construed as a potential conflict of interest.

## References

[B1] Baxter G. M. (ed.). (2020). Adams and Stashak's Lameness in Horses. Hoboken, NJ: John Wiley & Sons.

[B2] Burns E. N. Bordbari M. H. Mienaltowski M. J. Affolter V. K. Barro M. V. Gianino F.. (2018). Generation of an equine biobank to be used for Functional Annotation of Animal Genomes project. Anim. Genet. 49, 564–570. 10.1111/age.12717 30311254 PMC6264908

[B3] ENCODE Project Consortium (2012). An integrated encyclopedia of DNA elements in the human genome. Nature 489, 57–74. 10.1038/nature11247 22955616 PMC3439153

[B4] Fenner K. Caspar G. Hyde M. Henshall C. Dhand N. Probyn-Rapsey F.. (2019). It's all about the sex, or is it? Humans, horses and temperament. PLoS ONE 14:e0216699. 10.1371/journal.pone.0216699 31086385 PMC6516668

[B5] Furr M. Reed S. (eds.). (2015). Equine Neurology. Hoboken, NJ: John Wiley & Sons.

[B6] Garbe J. R. Da Y. (2008). User Manual Version 2.4. Minneapolis, MN: Department of Animal Science, University of Minnesota.

[B7] Hida N. Maeda Y. Katagiri K. Takasu H. Harada M. Itoh K. (2002). A simple culture protocol to detect peptide-specific cytotoxic T lymphocyte precursors in the circulation. Cancer Immunol. Immunother. 51, 219–228. 10.1007/s00262-002-0273-7 12012109 PMC11032804

[B8] Kingsley N. B. Kern C. Creppe C. Hales E. N. Zhou H. Kalbfleisch T. S.. (2019). Functionally annotating regulatory elements in the equine genome using histone mark ChIP-Seq. Genes 11:3. 10.3390/genes11010003 31861495 PMC7017286

[B9] Lee S. K. (2018). Sex as an important biological variable in biomedical research. BMB Rep. 51, 167–173. 10.5483/BMBRep.2018.51.4.034 29429452 PMC5933211

[B10] Li M. Wu H. Luo Z. Xia Y. Guan J. Wang T.. (2012). An atlas of DNA methylomes in porcine adipose and muscle tissues. Nat. Commun. 3, 1–11. 10.1038/ncomms1854 22617290 PMC3508711

[B11] Marras G. Gaspa G. Sorbolini S. Dimauro C. Ajmone-Marsan P. Valentini A.. (2015). Analysis of runs of homozygosity and their relationship with inbreeding in five cattle breeds farmed in Italy. Anim. Genet. 46, 110–121. 10.1111/age.12259 25530322

[B12] Raimondi E. Piras F. M. Nergadze S. G. Di Meo G. P. Ruiz-Herrera A. Ponsa M.. (2011). Polymorphic organization of constitutive heterochromatin in Equus asinus (2n = 62) chromosome 1. Hereditas 148, 110–113. 10.1111/j.1601-5223.2011.02218.x 21756256

[B13] Raudsepp T. Durkin K. Lear T. L. Das P. J. Avila F. Kachroo P.. (2010). Molecular heterogeneity of XY sex reversal in horses. Anim. Genet. 41(Suppl. 2), 41–52. 10.1111/j.1365-2052.2010.02101.x 21070275

[B14] Raudsepp T. Lee E. J. Kata S. R. Brinkmeyer C. Mickelson J. R. Skow L. C.. (2004). Exceptional conservation of horse-human gene order on X chromosome revealed by highresolution radiation hybrid mapping. Proc. Natl. Acad. Sci. U.S.A. 101, 2386–2391. 10.1073/pnas.0308513100 14983019 PMC356960

[B15] Sieck R. L. Fuller A. M. Bedwell P. S. Ward J. A. Sanders S. K. Xiang S. H.. (2020). Mandibulofacial dysostosis attributed to a recessive mutation of CYP26C1 in hereford cattle. Genes 11:1246. 10.3390/genes11111246 33105751 PMC7690606

[B16] Tsai H. W. Grant P. A. Rissman E. F. (2009). Sex differences in histone modifications in the neonatal mouse brain. Epigenetics 4, 47–53. 10.4161/epi.4.1.7288 19029819 PMC2667098

[B17] Will T. R. Proaño S. B. Thomas A. M. Kunz L. M. Thompson K. C. Ginnari L. A.. (2017). Problems and progress regarding sex bias and omission in neuroscience research. eNeuro 4:ENEURO.0278-17.2017. 10.1523/ENEURO.0278-17.2017 29134192 PMC5677705

[B18] Zucker I. Beery A. (2010). Males still dominate animal studies. Nature 465:690. 10.1038/465690a 20535186

