# Supplementary material for: Generation of a Biobank From Two Adult Thoroughbred Stallions for the Functional Annotation of Animal Genomes Initiative
Source: Front Genet. 2021 Mar 8;12:650305. doi: 10.3389/fgene.2021.650305 (PMC7982670; doi:10.3389/fgene.2021.650305)
Supplement: Supplementary file 1 [file Data_Sheet_1.pdf]

Table S1: List of tissues collected for two adult Thoroughbred stallions. Asterisk (*) denotes tissues with nuclei isolated.

<u>Musculoskeletal</u><br>Cartilage<br>• Stifle<br>• Thoracic metacarpo-phalangeal<br>• Pelvic metacarpo-phalangeal<br>Coronary periople (thoracic & pelvic)<br>Deep digital flexor tendon (thoracic & pelvic)<br>Superficial digital flexor tendon (thoracic & pelvic)<br>Suspensory ligament (thoracic & pelvic)<br>Gluteal muscle<br>Longissimus dorsi muscle*<br>Sacrocaudalis lateralis muscle<br>3rd Metacarpal/tarsal diaphysis<br>Rib bone marrow<br>Femur bone marrow<br>Sesamoid<br><br><u>Integument</u><br>Coronary periople (thoracic & pelvic)<br>Lamina (thoracic* & pelvic)<br>Hoof wall (thoracic & pelvic)<br>Neck skin<br>Unpigmented skin<br>Dorsum skin<br>Gluteal adipose tissue*<br>Abdominal adipose tissue<br>Loin adipose tissue	<u>Cardiovascular</u><br>Left atrium free wall<br>Left ventricle free wall*<br>Right atrium free wall<br>Right ventricle free wall<br>Aortic valve<br>Mitral valve<br>Pulmonic valve<br>Tricuspid valve<br><br><u>Respiratory</u><br>Trachea<br>Larynx<br>Cricoarytenoid dorsalis<br>Left lung*<br>Mediastinal/bronchiol lymph node*<br>Epiglottis<br><br><u>Endocrine</u><br>Thyroid<br>Adrenal cortex (left & right)<br>Adrenal medulla (left & right)<br>Pancreas<br><br><u>Urogenital</u><br>Kidney cortex (left* & right)<br>Kidney medulla (left & right)<br>Urinary bladder<br>Testis (left & right)<br>Right epididymis<br>• Caput<br>• Cauda<br>• Corpus<br>Bulbourethral gland<br>Prostate<br>Seminal vesicle<br>Ampulla<br>Ductus deferens<br>Corpus spongiosum<br>Corpus caversnosum	<u>Gastrointestinal</u><br>Liver (left lobe)*<br>Spleen*<br>Tongue<br>Esophagus<br>Stomach<br>Duodenum<br>Jejunum*<br>Ileum<br>Cecum<br>Right dorsal colon*<br>Right ventral colon<br>Left dorsal colon<br>Left ventral colon<br>Small colon<br><br><u>Nervous System</u><br>Dur mater<br>Parietal cortex*<br>Occipital cortex<br>Frontal cortex<br>Temporal cortex<br>Corpus callosum<br>Thalamus<br>Hypothalamus<br>Pituitary<br>Pons<br>Cerebellar vermis<br>Cerebellum lateral hemisphere*<br>Spinal cord<br>• C1<br>• C6<br>• T1*<br>• T8<br>Sciatic nerve<br><br><u>Eye</u><br>Cornea<br>Retina<br>Iris

**Table S2: Histopathology findings for two adult Thoroughbred stallions.**<br>Acronyms: no significant findings (NSF), pelvic limb (PL), thoracic limb (TL), cervical (C), thoracic (T), lumbar (L)		
Tissue	ECA_UCD_AH3	ECA_UCD_AH4
Skin Mid Neck	NSF	NSF
Skin Dorsum	NSF	NSF
Gluteal Adipose	NSF	NSF
Gluteal Muscle	NSF	NSF
Sacrocaudalis Muscle	NSF	NSF
Longissimus Dorsi Muscle	NSF	NSF
Right TL coronary band	NSF	NSF
Right TL & PL Lamina	NSF	NSF
Right PL suspensory lig.	NSF	NSF
Right PL Superficial Digital Flexor Tendon	NSF	NSF
Left TL & Right PL Deep Digital Flexor Tendon	NSF	NSF
Sciatic Nerve	NSF	
Liver	Increased bile ducts/oval cells; mild lymphocytic portal infiltrates;	Rare centrolobular and peripherolobular undefined round cell infiltrate and focal subcapsular fibrosis
Spleen	Active white pulp	NSF
Adrenal Cortex & Medulla	Markedly folded cortex; multifocal; vacuolar change of cortical cells	NSF
Kidney Cortex	NSF	NSF
Kidney Medulla	Mild lymphocytic infiltration	Mild lymphocytic infiltration
Larynx	NSF	Mild subepithelial lympho-plasmocytic infiltrate
Cricoarytenoid Dorsalis Muscle	Minimal interstitial lymphocytes	NSF
Pancreas	Sub capsular fibrosis	NSF
Thyroid	NSF	NSF
Mesenteric Lymph node	Marked cortical (active follicles) and paracortical hyperplasia	Cortical and paracortical hyperplasis
Trachiobronchiol Lymph Node	Marked cortical (active follicles) and paracortical hyperplasia	Cortical and paracortical hyperplasis
Left Lung	Multifocal fibrosis with mild lympho-plasmacellular infiltrates, contracted bronchi with lymphoid infiltrates	Perivascular lymphoid infiltrate; contracted bronchioles
Heart Left Atrium	NSF	Focal perivascular lymphoid myocarditis
Heart Left Ventricle	NSF; 4cm	Small aggregates of undefined round cells; 4.2cm
Heart Right Atrium	NSF	NSF
Heart Right Ventricle	NSF; 4.2cm	Perivascular and interstitial lymph-plasmacytic infiltrate in coronary fat/epicardium; 5.2cm
Ventricular septum	NSF; 4.1xm	NSF; 4.5cm
Mitral Valve	NSF	NSF
Tricuspid Valve	NSF	NSF
Aortic Valve	NSF	NSF
Pulmonic Valve	NSF	NSF
Trachea	NSF	NSF
Tongue	Minimal mutifocal perivascular lymphocytic glossitis	Minimal mutifocal perivascular lymphocytic glossitis
Epiglottis	Minimal lympho-plasmacellular infiltrates in lamina propria	NSF
Esophagus	NSF	NSF

Stomach	Lymphoytic gastritis with a lymph follicle formation	NSF
Duodenum	Mild enteritis	Mild enteritis
Ileum	Mild enteritis	Mild enteritis
Cecum	Lympho-plasmacytic and eosinophilic inflammation with prominent lymphoid tissue and lymphocytic exocytosis	Lympho-plasmacytic and eosinophilic inflammation with prominent lymphoid tissue and lymphocytic exocytosis
Right Ventral Colon	Lympho-plasmacytic and eosinophilic inflammation with prominent lymphoid tissue and lymphocytic exocytosis	Lympho-plasmacytic and eosinophilic inflammation with prominent lymphoid tissue and lymphocytic exocytosis
Left Ventral Colon	Lympho-plasmacytic and eosinophilic inflammation with prominent lymphoid tissue and lymphocytic exocytosis	Lympho-plasmacytic and eosinophilic inflammation with prominent lymphoid tissue and lymphocytic exocytosis
Left Dorsal Colon	Lympho-plasmacytic and eosinophilic inflammation with prominent lymphoid tissue and lymphocytic exocytosis	Lympho-plasmacytic and eosinophilic inflammation with prominent lymphoid tissue and lymphocytic exocytosis
Right Dorsal Colon	Lympho-plasmacytic and eosinophilic inflammation with prominent lymphoid tissue and lymphocytic exocytosis	Lympho-plasmacytic and eosinophilic inflammation with prominent lymphoid tissue and lymphocytic exocytosis
Small Colon	NSF	NSF
Eye (retina, cornea, iris, sclera)	NSF; retina missing	NSF
Frontal Cortex	NSF	Solitary perivascular lymphoid cuff
Occipital Cortex	NSF	NSF
Parietal Cortex	NSF	NSF
Temporal cortex	NSF	Perivascular lymphoid cuff
Pituitary	NSF	NSF
Cerebellum Vermis	NSF	NSF
Cerebellum lateral hemisphere	NSF	NSF
Pons	NSF	Perivascular lymphoid cuff
Thalamus	NSF	NSF
Hypothalamus	NSF	Focal meningeal lymphoid infiltrate
Dura Mater	NSF	NSF
Corpus Callosum	NSF	NSF
Spinal Cord C1	NSF	NSF
Spinal Cord C6	NSF	NSF
Spinal Cord T8	NSF	NSF
Spinal Cord L1	NSF	NSF
Spinal Cord L6	NSF	NSF
Dorsal Root Ganglia C4/5	NSF	NSF
Dorsal Root Ganglia T9/10	NSF	NSF
Urinary Bladder	NSF	NSF
Urethra	NSF	NSF
Prostate	NSF	NSF
Right Testicle	NSF; spermatogenesis present	NSF; spermatogenesis present
Left Testicle	NSF; spermatogenesis present	NSF; spermatogenesis present

Right caput epididymis		NSF
Right cauda epididymis	lymphocytic perivascular epididymitis	NSF
Right corpus epididymis	NSF	Minimal interstitial lymphocytic infiltrates
Seminal vesicle	NSF	NSF
Bulbourethral gland	NSF	NSF
Ampulla	NSF	NSF
Ductus deferens	NSF	NSF
Urethral process/corpus spongiosum	NSF	Lymphocytic balanitis
Body of penis/corpus cavernosum	NSF	NSF
Metacarpal III	NSF	NSF
Sesamoid	NSF	NSF

Table S3. a- and b- wave amplitudes and implicit times for scotopic electroretinography waveforms of two Thoroughbred stallions

	Horse	ECA_UCD_AH3						ECA_UCD_AH4					
	Flash stimulus (cd.s/m2) - white	0.01		3		10		0.01		3		10	
	Eye	OD	OS	OD	OS	OD	OS	OD	OS	OD	OS	OD	OS
a-Wave	amplitude (uV)	16.7	9.0	29.2	91.4	17.2	151.0	5.8	4.5	144.0	110.1	189.3	136.0
	implicit time (ms)	12.8	16.4	12.8	11.8	6.7	10.8	22.5	14.5	14.1	14.6	13.8	14.6
b-Wave	amplitude (uV)	230.8	186.8	358.1	200.8	614.0	375.8	245.6	305.7	364.8	498.1	411.4	444.7
	implicit time (ms)	83.5	91.6	38.9	35.3	69.6	65.5	76.8	67.8	57.7	58.5	61.4	59.2

Table S4: Epidydimal sperm post-thaw analysis for two adult Thoroughbred stallions

	ECA_UCD_AH3	ECA_UCD_AH4
Concentration (million/ml)	323.3	455.5
Number of straws		
Rapid progressive%	39.11	52.75
Slow progressive%	0.0	0.0
Non-progressive%	9.43	13.34
Immotile%	51.46	33.91
VCL (μm/sec)	110.2	100.5
VAP (μm/sec)	56.75	51.16
VSL(μm/sec)	43.03	36.59
LIN	0.39	0.36
STR	0.75	0.71
BCF (hertz)	26.23	22.58
ALH (μm)	4.28	4.49

	**Table S5: Synovial fluid analysis for two adult Thoroughbred stallions**					
	ECA_UCD_AH3			ECA_UCD_AH4		
	Left tarso-crural	Right middle carpal	Right radio carpal	Left tarso-crural	Left middle carpal	Left radio carpal
Gross appearance	Yellow/slight haze	Yellow/clear	Yellow/hazy	Yellow/clear	Yellow/hazy	Yellow/clear
Total protein (g/dL)	1.2	1.5	1.5	1.2	2.1	1.7
Mucin clot	Very good	Very good	Very good	Very good	Very good	Very good
Total RBC's (/μL)	<30,000	<30,000	<30,000	Rare	Rare	Rare
Total nucleated cells (/μL)	180	150	180	NA	NA	NA
Neutrophil %	0	1	2	0	1	0
Small mononuclear%	85	77	63	72	78	60
Large mononuclear%	15	22	35	28	21	40
Eosinophil%	0	0	0	0	0	0
Interpretation	No cytological abnormalities	No cytological abnormalities	No cytological abnormalities	No cytological abnormalities	No cytological abnormalities	No cytological abnormalities

	**Table S6: Cerebrospinal fluid analysis for two adult Thoroughbred stallions**	
	ECA_UCD_AH3	ECA_UCD_AH4
Gross appearance	Clear	Clear
Total protein (mg/dL)	72	88
Total RBC's (/μL)	6	<1
Total nucleated cells (/μL)	3	1
Neutrophil %	0	0
Small mononuclear%	95	85
Large mononuclear%	5	15
Eosinophil%	0	0
Interpretation	No cytological abnormalities	No cytological abnormalities

Table S7. Runs of homozygosity (ROH) and inbreeding estimate (F_{ROH}) for combined mare and stallion FAANG genome data sets

Length of ROH (Mb)	ECA_UCD_AH1	ECA_UCD_AH2	ECA_UCD_AH3	ECA_UCD_AH4
0 to 4	466	462	453	220
4 to 8	24	26	38	29
8 to 16	7	11	6	16

16 to 32	3	4	1	11
> 32	0	0	0	4
ROH Genome	765915572	838359933	758961216	1009849409
F_{ROH}	0.336	0.368	0.333	0.444

	**Table S8. Microsatellite genotyping of cryopreserved fibroblast cell line DNA from two adult Thoroughbred stallions compared to whole blood derived genomic DNA**			
	Type			
	Whole Blood	Fibroblast	Whole Blood	Fibroblast
Locus	ECA_UCD_AH3	ECA_UCD_AH3	ECA_UCD_AH4	ECA_UCD_AH4
AHT4	JK	JK	K	K
AME	YX	YX	YX	YX
ASB2	N	N	O	O
HMS3	I	I	MP	MP
HMS7	M	M	LM	LM
HTG4	K	K	KM	KM
LEX33	MQ	MQ	Q	Q
HMS2	L	L	L	L
AHT5	J	J	KN	KN
ASB17	GM	GM	NR	NR
ASB23	J	J	J	J
HMS6	MP	MP	MP	MP
HTG10	IO	IO	R	R
LEX3	H	H	N	N
VHL20	I	I	IL	IL

**Table S9. Color genotyping for two adult Thoroughbred stallions**		
	ECA_UCD_AH3	ECA_UCD_AH4
Red Factor	E/e	E/e
Agouti	A/a	A/a
Cream	N/N	N/N
Pearl	N/N	N/N
Silver	N/N	N/N
Dun	nd2/nd2	nd2/nd2
Champagne	N/N	N/N
Lethal White Overo	N/N	N/N
Sabino 1	N/N	N/N
Dominant White (W5, W10, W20, W22)	N/W20	W20/W20
Splashed White (SW1, SW3, SW5, SW6)	N/N	N/N
Splashed White (SW2, SW4)	N/N	N/N
Tobiano	N/N	N/N
Leopard	N/N	N/N
Patern-1	N/N	N/N
Gray	N/N	N/G
